# Demographics of road injuries and micromobility injuries among China, India, Japan, and the United States population: evidence from an age-period-cohort analysis

**DOI:** 10.1186/s12889-022-13152-6

**Published:** 2022-04-14

**Authors:** Yudi Zhao, Jinhong Cao, Yudiyang Ma, Sumaira Mubarik, Jianjun Bai, Donghui Yang, Kai Wang, Chuanhua Yu

**Affiliations:** Department of Epidemiology and Biostatistics, School of Public Health, Wuhan University, Wuhan, China

**Keywords:** Micromobility, Traffic fatalities, Road traffic injuries, Asia, United States

## Abstract

**Background:**

Micromobility sharing platforms have involved skyrocketing numbers of users in multiple countries since 2010. However, few studies have examined the overall impact of the growing micromobility market on road injuries.

**Method:**

We use road injury data from the Global Burden of Disease Study database to examine the effect of age, period, and cohort on micromobility injury-related deaths and incidence. We compared four countries that vary in demographic background and road infrastructure. By comparing the countries, we analyzed the relationship between the trends in road injuries and these factors.

**Results:**

We found an overall upward trend in micromobility injuries. A higher risk of micromobility-related injuries was witnessed in China and the US in 2015-2019, and people older than 45 showed a growing micromobility-related mortality and incidence rate in China, India, and the US. Cohorts after 1960 showed higher micromobility injury incidence risks in China and India, but the population born after 1990 in India showed a slightly lower risk compared to those before it.

**Conclusions:**

The boosted usage of micromobility devices explains these increasing trends. Road infrastructure and separated traffic ease the collisions from micromobility devices. The overall situation calls for improvement in legislation as well as road infrastructure.

**Supplementary Information:**

The online version contains supplementary material available at 10.1186/s12889-022-13152-6.

## Introduction

Micromobility is an innovative urban transport solution that provides flexible, sustainable, cost-effective, and on-demand transport alternatives. It aims at providing cost-effective short-distance travel solutions, solving the first or last-mile problem. In general definition, micromobility devices include motor scooters, powered two-wheelers, motorcycles, mopeds, bicycles, e-bikes, pedal-assisted bicycles, speed-pedelecs, mobility scooters, standing scooters, and e-scooters. Following 2010, numerous urban regions implemented shared micromobility, utilizing widely available Internet-enabled smartphones or other mobile devices, hastening the proliferation of micromobility devices [1]. Since 2017, China has had the largest micromobility platform [2]. In Japan, a similar robust industry for bike-sharing has developed over the last decade [3]. Since 1980, India has seen a rising number of registered two-wheelers [4]. With the rapid adoption of micromobility and shared micromobility services, the safety of micromobility devices has become an issue, despite the benefits of the alternative means of transportation in a city with excessive traffic. The Global Burden of Disease (GBD) estimated a 39.08 and 44.06% growth in motorcyclist and cyclist injury deaths since 1990, despite a 25.59% decline in overall road injury mortality.

Accidents involving micromobility have continued to rise in the US in recent years [5]. Since 1980, India has seen a rising number of fatalities caused by motorized two-wheelers, along with the rapid growth of registered two-wheelers [4]. In 2018, in a survey conducted on shared bike riders, 202 respondents out of the 2883 surveyed riders reported 292 bicycle-related road traffic injuries in China [6]. Studies warn that collisions tend to do severe damage to micromobility users. For instance, one study discovered that one in every ten e-scooter-and bicycle-motor vehicle collisions result in the motorcyclist’s or cyclist’s injury or fatality [7]. Additionally, hospitalization data supports that electric bicycle riders are at greater risk of enduring head injuries and require longer hospital stays than mechanical bicycle riders [8]. However, research suggests that safety regulations [9–11], road infrastructure [12, 13], and the legislative knowledge of the users [14] for micromobility are still lagging. Moreover, development is unable to keep up with the developing speed of the sharing system and the micromobility market.

In current research on micromobility, the lack of national standardization in micromobility data [7, 15, 16] makes it difficult to assess the overall impact. Numerous studies use data from emergency departments and trauma centers [7, 8, 10, 17], survey data [18], or news reports [5] to estimate the severity of micromobility collisions or their risk factors. However, such data is skewed toward more recent events and may reflect only a subset of the population’s injury trend. Furthermore, these studies cannot account for the effect of legislative changes on overall micromobility risks. As such, we seek to offer an overview trend of micromobility-related mortality from 1990 to 2019 and investigate the independent effects of chronological age, period, and birth cohort using the Age-Period-Cohort (APC) model, which was previously used to estimate road injury trends [19, 20] and is able to decompose the collinearity between age, period, and cohort. However, road injuries are often related to other factors such as overall road infrastructure style, culture differences, and driver habits. Therefore, in this research, we selected four countries that are diverse in these factors: China, India, Japan, and the United States (the US). By weighing the age, period, and cohort effects between countries, we can see how these factors affect the shifts in micromobility-related deaths.

China and India are both middle-income countries. Both countries are snowballing in both population and motor vehicle numbers. India has been highly reliant on motored two-wheelers since 1980 [4], while China has been dominated by bicycles for decades [21]. Though both countries invest heavily in improving road infrastructure, the governments have shown different priors on road width or intersections, highways, or rural roads. We may expect China to witness a tamer reaction to the rapid growth of micromobility device usage due to its heavy reliance on bicycles. The reaction of the period effect may show how infrastructure development may help ease road injury. Japan and the US are both high-income countries. Both have well-developed road infrastructure and a long automobile history. However, Japan has fewer registered autos per capita in urban areas than the US [22]. Legislation in the US varies between states, while the legislation is primarily applicable in different prefectures in Japan. Also, the Japanese tend to believe traffic accidents are “theirs to blame” and think of these accidents as a severe problem, while Americans tend to take them less seriously [23]. The differences in age effects may show how culture affects the same age groups. Furthermore, the cohort effect may correspond with the road infrastructure development legislation. The findings of this study will aid in the development of effective micromobility and overall road safety regulations.

A comprehensive understanding and up-to-date analysis of road injury and micromobility-related mortality and incidence are required to comprehend how present regulations, road infrastructure, and road users cope with the growing use of micromobility. To address this knowledge gap, we look at the overall trend and the age, period, cohort effect of road injury and micromobility-related injury deaths and incidence in China, India, the US, and Japan. We investigated the effects of legislation, road infrastructure, and road user characteristics on road injury and micromobility-related injury by comparing the impacts and trends over time and across nations.

## Methods

### Data sources

The analysis is based on the Institute for Health Metrics and Evaluation’s most recent Global Burden of Disease database (GBD2019) [24]. The GBD 2019 aims to quantify the comparative magnitude of health loss due to diseases, injuries, and risk factors by age, sex, and geography for specific locations. The injury was estimated using primary data sources, including vital registration, verbal autopsy, and police records. Detailed original data sources for each country are listed on the GBD input source website (http://ghdx.healthdata.org/gbd-2019/data-input-sources). The data was processed via the cause of death estimation model. The cause of death was established using redistribution algorithms, and unspecified injuries were redistributed based on the causal chain in which the ICD codes X59 (“exposure to an unknown factor”), Y34 (“unspecified incident, unknown intent”), and GBD injury causes were the underlying causes of death. After processing the data, estimates of each quantity of interest were generated by age, sex, location, and year. The modeling standardization was done using the Cause of Death Ensemble model (CODEm), spatiotemporal Gaussian process regression (ST-GPR), and DisMod-MR. Detailed data processing and model covariates are available in Abbafati et al., 2020. The age-standardized mortality and incidence rates are standardized to the GBD 2019 global age-standard population. The calculation formulas for age-standardized mortality rate (ASMRs) and age-standardized incidence rate (ASIRs) are as follows:

$$\mathrm{ASMR}=\frac{\sum \mathrm{Age}\mathrm{composition}\ \mathrm{of}\ \mathrm{standard}\ \mathrm{group}\ \mathrm{population}\times \mathrm{Age}\ \mathrm{specific}\ \mathrm{mortality}}{\mathrm{Age}\ \mathrm{composition}\ \mathrm{of}\ \mathrm{standard}\ \mathrm{population}}$$

$$\mathrm{ASIR}=\frac{\sum \mathrm{Age}\mathrm{composition}\ \mathrm{of}\ \mathrm{standard}\ \mathrm{group}\ \mathrm{population}\times \mathrm{Age}\ \mathrm{specific}\ \mathrm{incidence}}{\mathrm{Age}\ \mathrm{composition}\ \mathrm{of}\ \mathrm{standard}\ \mathrm{population}}$$

Most countries consider micromobility devices to be motorcycles or bicycles based on whether the model exceeds the maximum speed limit for micromobility devices. For example, China considers all e-bikes that meet the speed and weight standards to be bicycles. Additionally, the ICD-10 code does not apply to micromobility-related injuries before October 2020 [15]. Therefore, we obtained the population, mortality, fatality, ASMRs, and ASIRs data caused by road injuries, motorcycle road injuries, and cyclist injuries in China, India, Japan, and the US from GBD 2019. We consider motorcyclist injuries and cyclist injuries to be micromobility injuries. In the GBD, road injuries encompass injuries involving motor vehicles, pedestrians, motorcyclists, and cyclists. Motorcyclist injuries include motorcycle riders injured in transport accidents; cyclist injuries include pedal cyclists injured in transport accidents (detailed ICD codes can be seen in supplementary Table 1).

### Statistical analysis

When estimating mortality, standard statistical analysis could not decompose the death risks and health risks. The APC model could estimate the cumulative health risks for the past birth cohort and show the mortality risk for the population within each period. Road injuries are highly relevant to a country’s economic status, regulations, and road infrastructure. Moreover, age is an essential factor, impacting both the legislation and the related risk factor in traffic collisions. Therefore, the APC analysis decomposed time trends and provided relatively efficient estimation results. As the birth cohort was calculated using period and age (birth cohort = period - age) [25], the relationship between them is perfectly linear. Therefore, we avoid this problem by producing estimable APC parameters and functions without imposing arbitrary constraints on model parameters [26].

In this analysis, age reflects changes in vital rates; the risk of incidence increases with age. In the aspect of the age effect, the net drift denotes the annual percentage change of the expected age-adjusted rates over time, while the local drifts represent the annual percentage change of the expected age-specific rates. The longitudinal age curve can show the reference cohort’s age-specific mortality rates. In the aspect of time effect, the period/cohort RRs imply the relative risks of mortality or incidence in each period/cohort compared to the reference one. The period effect may be affected by historical events and environmental factors. In contrast, cohort effects are affected by uneven population exposure or unequal exposure levels in different age groups at a critical development period.

The mortality and incidence rates were age-standardized based on the GBD 2019 global age-standard population [27]. The age-specific mortality rates between 1990 and 2019 were ranked into consecutive 5-year periods, starting from the age group of 5-9 years and ending with the age group of 75-79. Since road injury occurs in every age group, we took the central group -- the 40-44 age group as the reference group for better comparison. The period 2000-2004 was used as a baseline, with the 1960-1964 cohort serving as a reference accordingly. We adopt the APC Web Tool for parameter estimation along with associated statistical hypothesis tests [28]. The Web tool adopts the ID approach of the APC model and is an open-sourced program based on the R language. Its core operation code is detailed on GitHub (https://github.com/CBIIT/nci-webtools-dceg-age-period-cohort). The equations used in the model are as follows [28]:

Longitudinally, the expected rate per 100,000 person years among persons born in year *c* and followed-up at age *a*: *R*(*a*| *c*) = *LongAge*(*a*| *c*_0_) × *CRR*(*c*| *c*_0_) × *e*^*CD*(*c* + *a*.)^

Cross-sectionally, the expected rates by age conditional on period: *R*(*a*| *p*) = *CrossAge*(*a*| *p*_0_) × *PRR*(*p*| *p*_0_) × *e*^*CD*(*p* − *a*)^

Expected rates by period conditional an age: $R\left(p|a\right)= FTT\left(p|{a}_0\right)\times \frac{CrossAge\left(a|{p}_0\right)}{CrossAge\left({a}_0|{p}_0\right)}\times {e}^{CD\left(p-a\right)}$

This study used a specific set of estimable functions to conduct the APC analysis. Wald chi-square tests were carried out to verify the significance of estimable parameters and functions. Ethical approval was not needed for this study because there was no direct involvement of human subjects.

## Results

### General trend

**Fig. 1:**
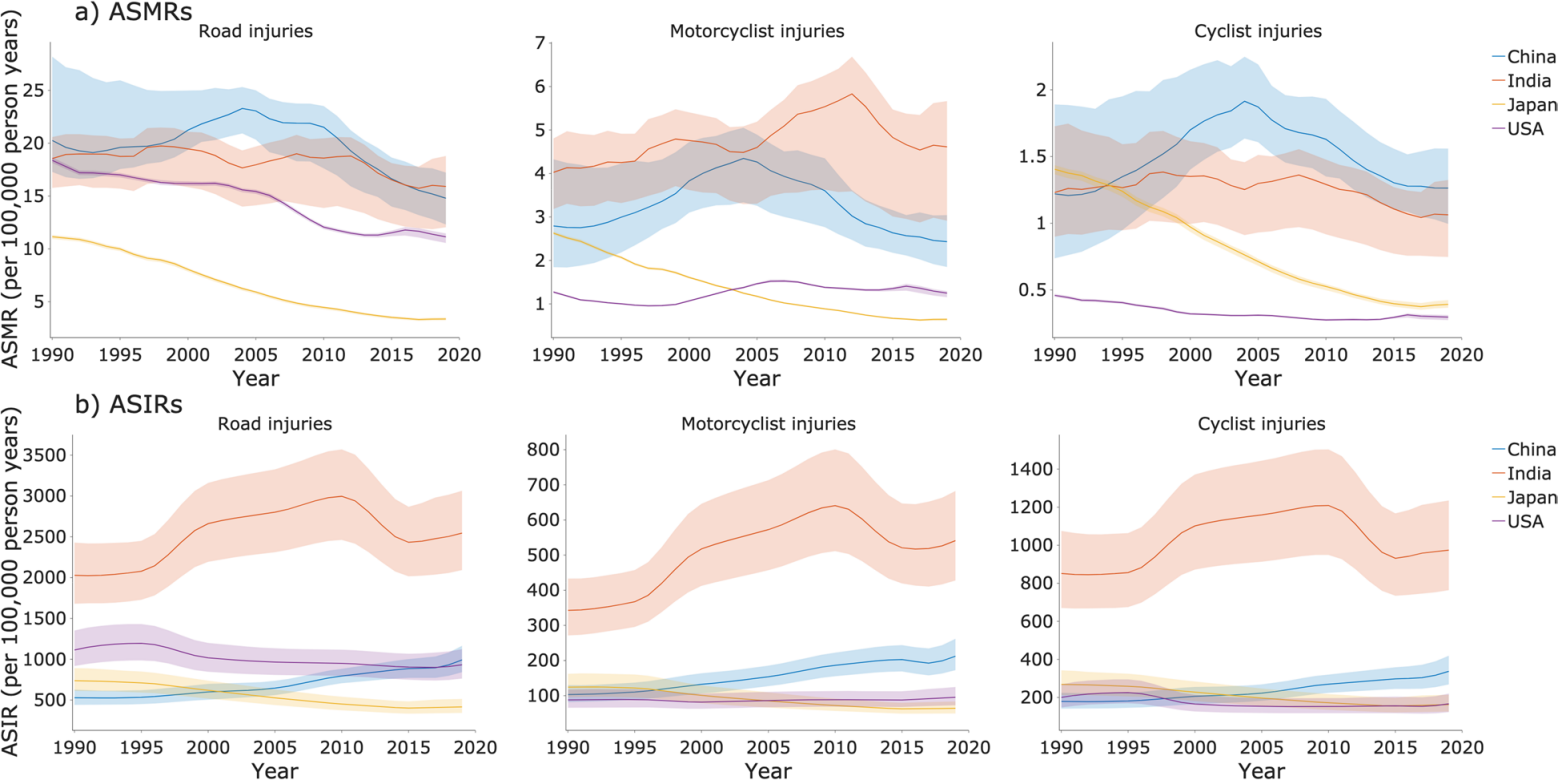
Trends of the (**a**) age-standardized mortality rates (ASMRs) and (**b**) age-standardized incidence rates (ASIRs) per 100,000 population for road injuries, motorcyclist injuries, and cyclist injuries in China, India, Japan, and the US, 1990 to 2019

Figure 1 shows the ASMRs and ASIRs trends per 100,000 population for road injuries, motorcyclist injuries, and cyclist injuries in the US, Japan, India, and China. The ASMRs of road injuries (Fig. 1a) in China were higher than in all three other countries from 1990 to 2016, at 20.259, with a first increasing and then declining trend. Motorcyclist injury ASMRs have an overall higher rate than cyclist injury ASMRs. Motorcyclist injuries in India are the highest among the four countries, accumulating from 1990 to 2012, peaking at 5.830 and gradually declining to 4.541 until 2017, then inclined slightly afterward. In Japan, road injuries, motorcycle road injuries, and cyclist injuries have shown a clear downward trend until 2018.

The ASIRs of road injuries (Fig. 1b) in India were almost two-fold higher than in all the other three countries in 1990 and rapidly grew to three-fold in 2010. China showed a clear upward trend in the past three decades, and the trend showed a rapid acceleration in recent years. The US showed a slight incline from 1990 to 1995, then mildly declined until 2017. Japan has shown a tame declining trend since 1990. Motorcyclist injury ASIRs and cyclist injury ASIRs showed a similar trend with road injury ASIRs, with motorcyclist injury ASIRs being lower than cyclist injury ASIRs.

### APC analysis

**Fig. 2:**
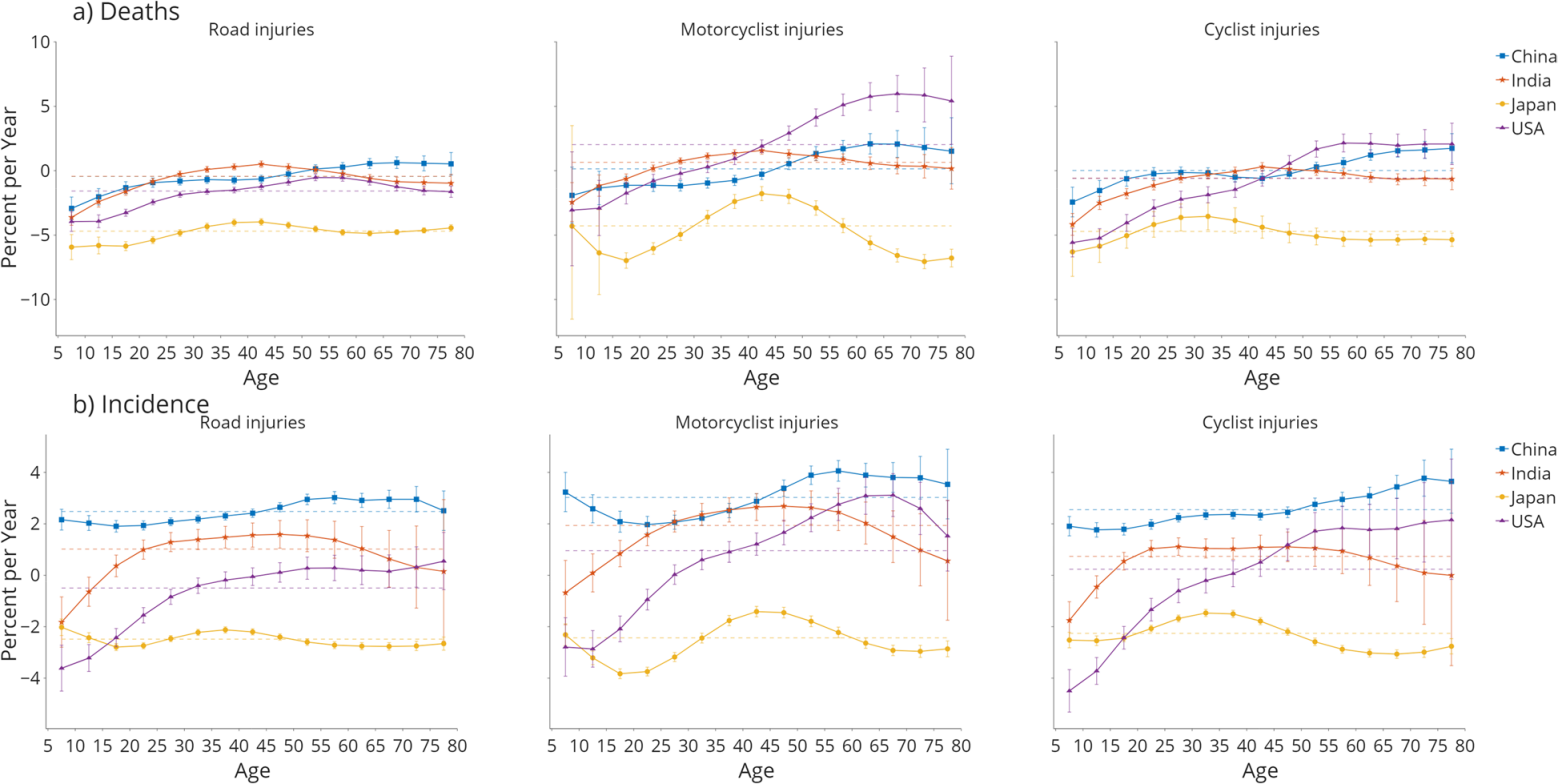
Age group-specific annual percent change (local drift) with overall annual percent change (net drift) values for road injury, motorcyclist injury, and cyclist injury (**a**) mortality and (**b**) incidence of in China, India, Japan, and the US. Net drift values are depicted as dotted lines. Error bars represent the 95% CIs for the local drift values

Figure 2 shows the net and local drifts for the mortality and incidence of road injuries, motorcyclist injuries, and cyclist injuries in the four countries. The net drift indicates the annual percentage change of the expected overall age-adjusted rates. Figure 2 depicts the net drift per year for road injury-related deaths and incidences (supplementary Table 3 for detailed data). The net drift for the incidence rate suggests a much worse situation, especially for motorcyclist injury incidences. The local drifts bespeak the expected age-specific rates over each age group. Only the US and Japan have a local drift totally below zero among all age groups for road injury deaths. China and India witnessed a sharp increment since age 5, exceeding zero at age 30 for India and 50 for China. For motorcyclist injuries, all countries showed an increment from age five, except for Japan, where the local drift dropped from age 5-19 and then increased until age 40-44. Only Japan has local drifts below zero for all age groups for motorcyclist injuries and cyclist injuries among all four countries. Both China and India peaked at 65-70, with India reaching 5.976% and China at 2.070. The local drift shows a spiked risk after age 5 in all four countries for cyclist injuries. Japan reached the highest local drift in the age group of 30-34 at − 3.547%, while India topped 55-60 at 2.147%. China showed a camel curve where the local drifts reached a high point in the age group 25-30, exceeded zero after age 50, and continued to accumulate for all older age groups.

The local drifts in road injury incidence are generally increasing in China, India, and the US. The age group of 20-59 sees the highest local drifts for Indians. In the US, the sharpest increment with age occurred after age 45. The local drifts for motorcyclist injury incidences showed a more dynamic trend, with only Japan having all age groups below zero. China first decreased for age groups under 25, then increased and peaked at 4.058% for the age group 55-59. The US showed an even steeper trend, exceeding zero at age group 25-29 and peaking at 3.115% at age group 65-70. The wavy trend in Japan is consistent with road injury incidence. The incidence of cyclist injuries showed a similar trend to that of road injury incidence.

**Fig. 3:**
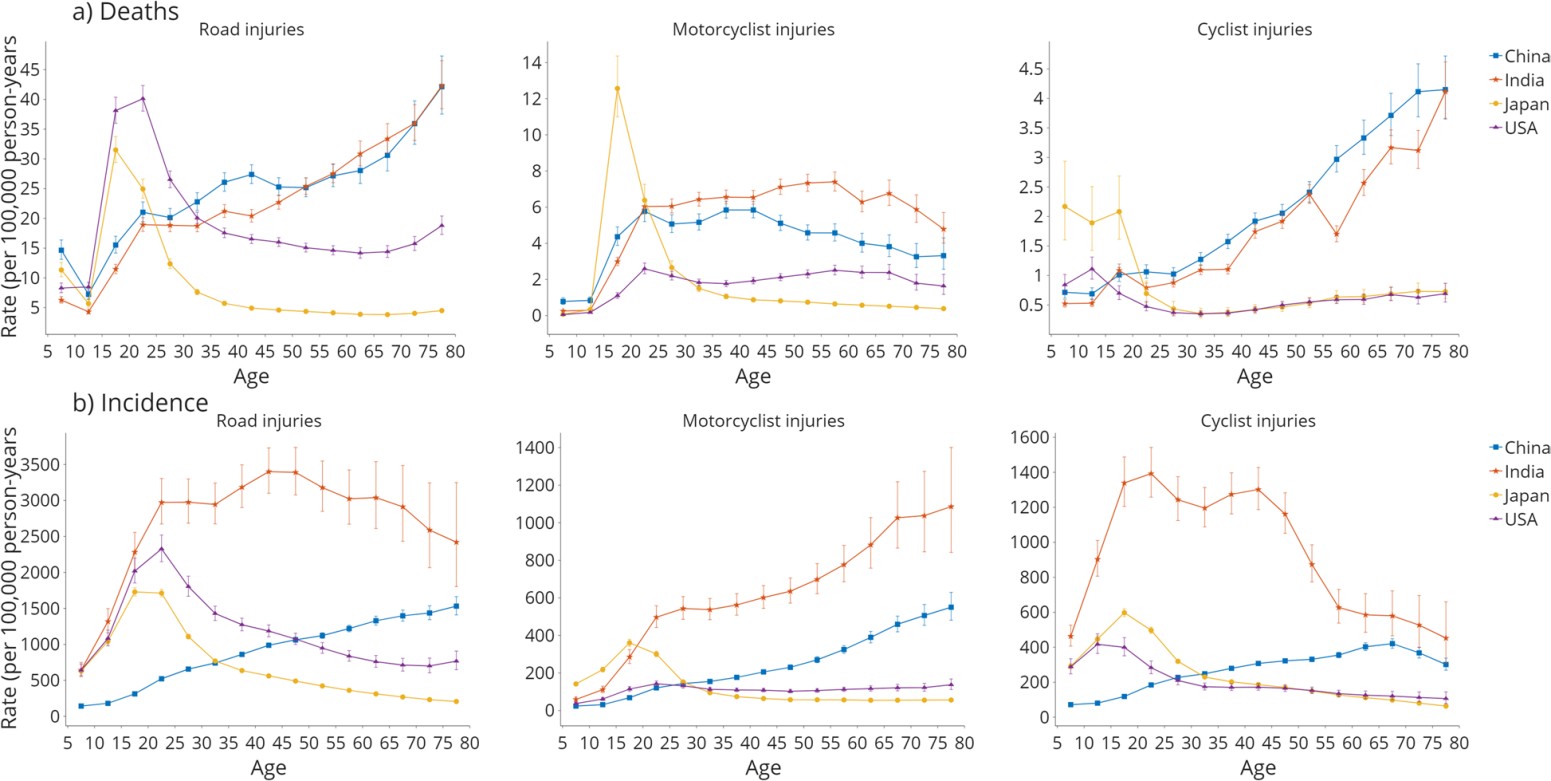
Fitted longitudinal age-specific rates of (**a**) mortality and (**b**) incidence (per 100,000 person-years) caused by road injury, motorcyclist injury, and cyclist injury in China, India, Japan, and the US. Error bars represent the 95% CIs for the longitudinal age curve values

The longitudinal age curves showed how risk differs between ages in the same birth cohort. For the road injury death rate (Fig. 3a), all four countries showed a spike in 15-19, with the US having the highest at 38.115. Unlike the US and Japan, which showed a lower rate for older age groups, China and India’s rates escalated while aging. Motorcyclists’ injuries and deaths showed a different trend. The 15-19 age group showed significant growth among all four countries, with Japan having the highest at 12.570. China, India, and the US showed a relatively stable growth trend with age. Cyclist injury mortality showed a similar trend to the road injury death rate, while Japan showed a higher death rate among age groups under 20.

For the road injury incidence rate, all four countries showed an increasing risk from 5 to 24. However, Japan and the US showed a downward incidence rate trend after age 25, while India and China showed higher incidence rates for older age groups. For the motorcyclist injury incidence rate (Fig. 3b), Japan peaked at the age group of 15-19 and rapidly declined. China and India see a similar trend in road injury incidence rates, with India seeing a more evident incline trend than China. The cyclist injury incidence showed a higher risk for age groups before 20 in Japan, the US, and India, then rapidly decreased for older age groups in Japan and the US. In China, the incidence rate accumulated until 70, then mildly dropped to 70-79.

**Fig. 4:**
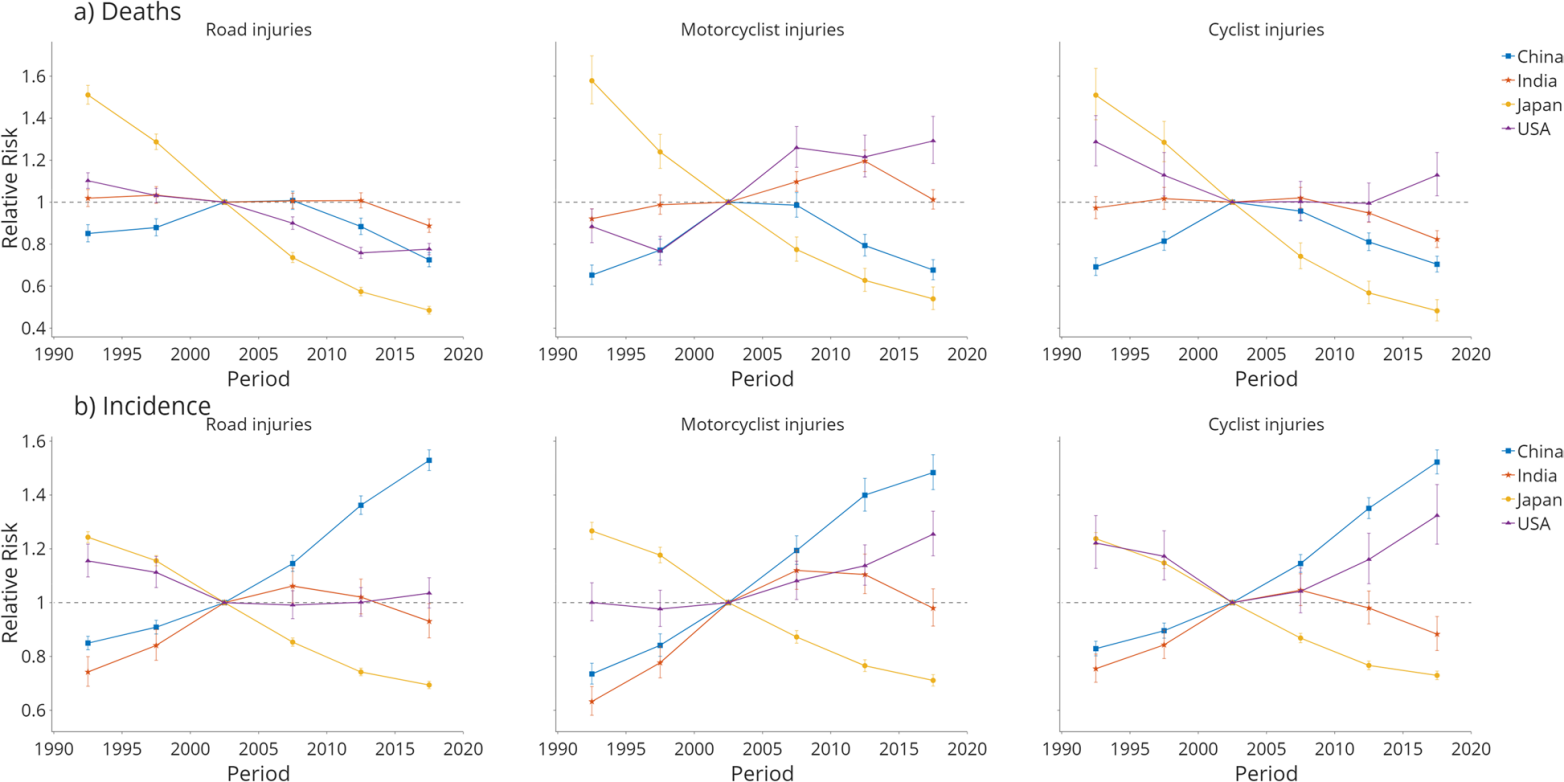
Period relative risks (RRs) of (**a**) deaths and (**b**) incidences for road injuries, motorcyclist injuries, and cyclist injuries in China, India, Japan, and the US. The relative risk of each period is compared with the reference period (2000-2004). Error bars represent the 95% CIs for the period relative risks. Relative risk = 1 is added for each graph as reference

**Fig. 5:**
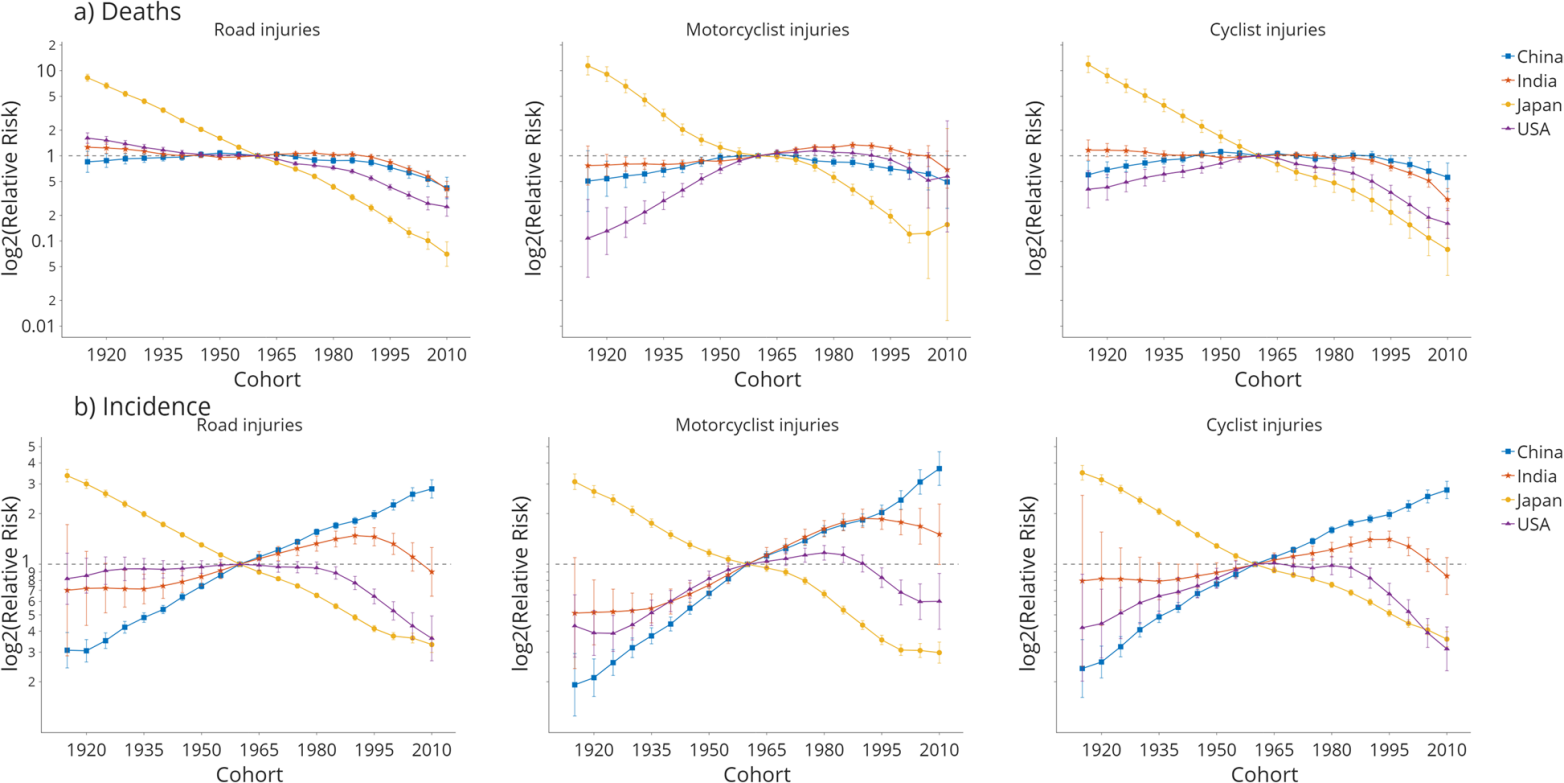
Cohort relative risks (RRs) of (**a**) deaths and (**b**) incidences for road injuries, motorcyclist injuries, and cyclist injuries in China, India, Japan, and the US. The relative risk of each cohort is compared with the reference cohort (1960). Error bars represent the 95% CIs for the period relative risks. Relative risk = 1 is added for each graph as reference

The estimated period and cohort RRs by country are shown in Figs. 4 and 5. The period RRs of road injury deaths (Fig. 4a) showed a generally downward trend. However, only Japan showed a clear downtrend for all injury deaths from 1990 to 1994 to 2015-2019. Period RRs increased from 1990 to 2009, then decreased in China. The US declined from 1990 to 2014 but witnessed an increment in the most recent period. For motorcyclist injuries, China and Japan showed a similar trend to road injuries. In contrast, India showed a higher RR in the period 2010-2014, and the RR of motorcyclist deaths in the US continued to rise since 1995, reaching 1.291 in the most recent period. The cyclist period RR showed a similar trend with road injuries in China, Japan, and India, while the US showed the first decrease, then an increase trend.

For all three types of injury incidences (Fig. 4b), the period RRs showed a downward trend for Japan and a clear upward trend for China. India showed an upward trend from 1990 to 2010, then a downward trend afterward. The US experienced improvement before 2005, then worsened in the periods after for all three types of injuries.

The cohort RRs showed generally decreasing road injury patterns among all countries, indicating a more substantial reduction in road injury mortality among younger generations regardless of the age and period effects. The cohort RRs witnessed a drop in all four countries for road injury deaths (Fig. 5a), where Japan witnessed the most significant decline. Motorcyclist injury cohort RRs showed a pronounced decline in Japan and an incline after 2000. China and India witnessed an increment in cohorts before 1985, then decreased for recent cohorts. Cyclist injury deaths showed a similar trend to road injury deaths, while China and the US increased for cohorts before 1960. For the incidence RR (Fig. 5b), Japan showed a clear downtrend for all three types of injuries, and the trend was reversed in China. India showed an upward trend for cohorts before 1990, while the US started to show a decreasing trend after 1980.

The Wald Chi-Square tests for estimable functions in the APC model are presented in supplementary Table 2.

## Discussion

Our findings revealed a drop in traffic injury fatalities and incidents during the last three decades. In general, Japan and the US saw fewer fatalities and incidents than India and China. However, fatalities and incidences of micromobility injuries have increased in recent years across the four countries. Our APC analysis demonstrated a more significant death and incidence rate associated with micromobility in the under-25 and over-60 age groups. In recent years, local drifts have also demonstrated a growing trend in the senior population’s mortality and incidence rates. Additionally, whereas road injury mortality risks have decreased over the last three decades, motorcyclist deaths and occurrences have grown in recent decades and birth cohorts.

The ASMR of road injury mortality in China was the highest among the four countries, increasing from 1990 to 2005 and rapidly decreasing after implementing its first law on Road Traffic Safety in 2004. However, owing to the heavy reliance on motorcycles, India showed higher ASMR than China in motorcyclist injury deaths, increasing until 2012. This declining time point corresponds to the rural road network development achievement under the Bharat Nirman program [29]. China and Japan showed relatively higher ASMR in cyclist injuries than the US and India, matching the popularity of bicycles in both countries [21, 30]. Yet the rapid decline of ASMR in Japan showed that separated motorized and non-motorized road infrastructure helps significantly decline cyclist injury mortality.

The ASIR among the three types of wheeled-device injuries showed a similar trend. India ranked first among the four countries in all three types of incidence rates, with an increasing trend. Though the rural road infrastructure has gone through multiple development plans since 1943, the separation of different types of vehicles is still poor in urban areas and highways [31, 32]. Though China has invested much more in road infrastructure development compared to India, the incidence rate of the three types of injuries is still increasing and has doubled in China during the past two decades. This could be explained by the speed of vehicle growth in China, which has been 10-fold since 1990, compared to only three folds in India [21]. The US and Japan showed a relatively high incidence rate of road injuries compared to motorcyclist and cyclist injuries, proving that separated motor and non-motor roadways help decrease collisions.

The age effect of road injury mortality showed that age groups 10-24 have a sharp rise in mortality rates in all four countries. This finding is consistent with other research suggesting that adolescent drivers behave in unsafe ways when operating motor vehicles and micromobility devices [33–36]. This characteristic is applicable to different demographic backgrounds. The earlier access to a driver’s license in the US may explain the abrupt increment in the age group of 15-19. The older age groups showed a lower mortality rate in Japan and the US, yet this does not mean that the growing micromobility market has little impact on the older age groups in the US and Japan. Though Japan had all age groups under zero, indicating an overall declining rate for road injuries, the older age group showed a slower descending trend than the other age groups. The US local drift for road injury deaths is generally under zero for all age groups, yet the local drift of motorcyclist and cyclist deaths soared above zero for age groups after 45. This trend was even more pronounced in micromobility-related injury incidences, indicating that the older population has been exposed to an increased risk of road injury in recent years. Questionnaire-based research found that e-bike users are mostly older populations (> 56), and middle-aged groups (36-55) use conventional bikes in the US [33]. The older population prefers e-bikes over traditional bicycles for assistance going uphill and to further destinations [34]. These may become the determining factors for the accumulating tendency. Additionally, since e-bikes can go faster than traditional bicycles, speed is found to impact the occurrence of collisions [37].

Unlike in Japan and the US, where the fatality rate decreased with age, mortality climbed with age in China and India. Also, most age groups in China and India showed a local drift above zero, indicating an overall growth in traffic collisions in all age groups, also reflected in the period and cohort RRs. The disparity between high- and middle-income countries may be partly explained by the effectiveness of their post-crash emergency response [16]. However, it is worth noting that in India, the local drifts for motorcycle fatalities and occurrences in school-aged children are negative. Underage motorbike use has been a significant issue [38]. Local drifts demonstrate that the motorcycle registration requirement is having an impact gradually.

The age effect of motorcyclist injury mortality showed a growing trend for age groups after 15 among all four countries and remains high for China and India. Research in China discovers that younger generations and senior populations with poor education levels exhibit erroneous conduct when using e-bikes [36, 39], while in India, there was reported underage usage of motorcycles [38]. This is also consistent with reported road injury fatalities between 18 and 49 [12]. A high death rate was witnessed in Japan among the 15-19 age groups. This may be explained by the early access to a motorcycle license at age 16. Cyclist injury mortality in Japan showed a high death rate among age groups before 20. In Japan, students often rely on bicycles for transit. Though school routes are provided around schools to prevent collisions involving school-aged children [40], students who work part-time or take additional studies tend to ride bicycles and are not protected by special pathways. The cyclist mortality rate continues to rise with age in China and India, in contrast to Japan and the US. This indicates that post-crash emergency response and separation of mixed traffic is one major issue in developing countries.

The age effect of the incidence rate of road injuries showed higher risks for age groups between 15 and 25 in all three countries except China. Prior research in India showed similar results, reporting a higher incidence rate in the 18–39 age group [12]. The age-effect of the incidence rate in Japan and the US showed a similar trend to the mortality rate, which indicates that the risky behaviors among young road users are often fatal. In China, the incidence rate grows with age. Past research states that China’s being new to motorized devices means being unfamiliar with the device may cause erroneous reactions on the road and cause collisions [36, 41]. In both India and China, the incidence of motorcyclist injuries increases with age. In India, cyclist injury incidence showed higher rates in age groups before 60. When combining this trend with the mortality age effect in the cyclist injury incidence rate, we noticed that while younger age groups have a higher incidence rate in India, older age groups have a higher mortality rate. This indicates that cyclists of all ages in India are exposed to higher risks of collisions and that these collisions are often fatal to older age groups. Additionally, as we explored the local drifts for mortality, we discovered that India’s 20-59 age groups had a growing tendency over the previous decade. This may indicate that adolescents and middle-aged individuals are becoming victims of road injuries, particularly motorcycle accidents, with the most significant annual increase rate. Furthermore, the data sources in the previously stated report bolstered our speculation, given that the report’s majority of research is based on hospital data and utilizes recent records [12].

Period effects of road injury mortality showed a generally declining RR among the four countries. This signifies that the effort on vehicle standards, road infrastructure, overall medical care, and traffic regulations is effective. All three types of injury-related deaths and incidence risks showed a continuously decreasing trend in Japan, particularly after implementing a strict drunk driving regulation in 2002 [42]. These trends indicate a considerable improvement in the overall state of road injuries in Japan. Since the Road Traffic Law modification in 2004 to prohibit cell phone use while driving, injury crashes have decreased consistently, complying with our results. Road injury deaths RR has also dropped in the US. Since 1984 when New York State established seatbelt laws, seatbelt usage rose from 12% to 50 from 1985 to 1986, and rose to 82% in 2005 and 86% in 2012 [19]. China sees an increase, then a decrease in RR, with a turning point between 2000 and 2005. China did not have its first traffic laws until 1988, when the Rules of Urban Traffic were released. However, this aims to improve road traffic management and maintain traffic order to satisfy the needs of modernization rather than to ensure road user safety. It was not until 2004 that China implemented its first law on Road Traffic Safety. In 2009, special enforcement of drunk driving and seatbelt regulations was initiated, and amendments were made to address emergency traffic management, traffic security specifications, construction standards, and violation processing. These rapidly developing regulations explain the strong increasing trends before 2010 and the accelerated decline. India witnessed a boost in period RRs after 2005. Prior to 2006, most urban roadways were narrow and unpaved, mostly lacking pavement and cycle lanes. Congestion and safety issues on the roads are exacerbated by the narrow roads, over-occupied rickshaws and motorcycles, and uncontrolled on-street parking [21]. The drop after 2005 corresponds with the project launched in 2006 to improve the quality of roadways and intersections. Period RR in motorcyclist injury mortality showed a growing tendency in India and the US. This reverse incline in the US, along with the incline in period RR in cyclist injuries, is consistent with the expanding demand for micromobility devices. Shared bicycles started in 2012 in Washington, DC, and e-scooters were first introduced in 2017 in Santa Monica, CA. By the end of 2018, cities across the US had deployed over 57,000 shared bicycles and e-bikes and 85,000 e-scooters. Until 2018, riders made a combined 36.5 million journeys on bicycles and e-bikes and 84 million trips on e-scooters [43].

The motorcyclist injury mortality period effect in India has maintained an RR above one until the most recent. For the last decade, India has been working on two-wheeler laws. Research suggests that two-wheelers had the highest number of violations filed in 2000 [44]. In 2015, they strengthened helmet-wearing legislation by requiring helmets to be attached. Nevertheless, according to the same year’s research, India’s estimated helmet-wearing rate remained low at 30% for drivers and over 10% for passengers.

Additionally, in 2018, a new motorbike ABS standard was implemented, aiming to force motorbikes to apply ABS to ensure the driver’s safety [16]. Cyclist injury period RR showed a similar trend to road injury mortality. However, India has shown increased risk in recent periods. This is also the result of mixed traffic on rural roads [45] and the lack of a bicycle riding culture [21] compared to China. The period effect in incidence risk showed a similar trend to mortality. However, China sees a continuous rise in all three types of injuries. Road infrastructure, historical traffic cultures, and growing motorization could explain the persistent upward tendency [41].

Cohort RRs exhibit similar trends to period RRs, yet they reveal longer trends among the four countries. The overall cohort RRs for mortality showed a declining trend in all four countries, indicating that the familiarization of motorized culture, the development of legislation, and road infrastructure contributed to decreasing the mortality rate. However, the cohort effect showed higher RRs in road injury incidences. The rapid decrease of cohort RRs in road injuries results in the fast response of Japanese road infrastructure. Before 1950, the road system was noted as “incredibly bad.” However, after the release of the Highway Public Corporation Law in 1956, public works expenditures tripled between 1960 and 1970 and continued until 1990. This achieved a world-class, technologically advanced road system. Since 1970, when the Traffic Safety Policy Law was enacted, pedestrian fatalities caused by inadequate space between autos and other traffic users have been cut in half. Following that, the law was revised every 5 years [41]. In the US, the cohort RR reveals a gradual decline after 1960 and a steep decrease after 1990. Cohorts prior to the 1960s witnessed a shift from head company-funded highways to state-funded projects. This accounts for the elevated RRs observed in cohorts around 1960. It was followed by the National Traffic and Motor Vehicle Safety Act in 1966 to alleviate the massive rise in death rates caused by it [41]. The cohort effect in the road injury incidence rate showed a higher risk in China and India for cohorts after 1960. Despite the Chinese government’s extensive investigation into extending urban roadway networks [21], China observed poor infrastructure design, such as narrower spacing between urban expressway entrances, excessive signal distances [41], and unsafe crossings [21]. In India, the cohort effect continuously increased and peaked around 1995. Road infrastructure development in India was halted between 1947 and 1997 but accelerated following the passage of the National Highway Act, which corresponds to the time when cohort RR decreased.

For the cohort RRs among motorcyclist injuries in the US between 1990 and 2005, the rapid increase in motorcycle sales and the lack of uniformity in insurance, age, license, and training regulations throughout the US [46] may have contributed to the cohorts’ high RR in motorcyclist injuries between 1980 and 1990. India sees increasing risk for recent cohorts, corresponding to the significant growth in registered motorized two-wheelers. Two-wheelers in India have vastly exceeded cars since. 1980 [4] Though the road infrastructure has improved during the past two decades, the government’s primary objective is to create expressways linking cities rather than urban roadways. The roadways in urban cities are still over-occupied and lack separation between motor vehicles and other road users. The risk of both motorcyclist and cyclist injuries has shown continuous growth in China. This indicates that younger generations are more reliant on motorcycles and mopeds, yet they still lack road safety knowledge. Also, road infrastructure and regulations may require a new revision. Though China has a strong cycling culture, and most cities have bicycle lanes, the infrastructure is far from adequate to accommodate the high volume of walking and cycling excursions. The quality also lags far behind that of northern European countries [21]. Moreover, the relative youth of traffic laws or driver inexperience [35, 46] as well as “scrambling” behaviors among automobiles, pedestrians, and micromobility devices [47], led to unsafe roadways. In industries such as food delivery and parcel delivery, wearing a helmet during delivery is mandatory [14]. However, the helmets could not prevent collisions. In addition, the personnel in these industries tend to “scramble” due to the limited time of orders.

We are surprised to see that the rising micromobility industry has had little effect on Japan’s overall decline in motorcycle and cycling injuries. Firstly, Japan has a well-developed roadway system due to the significant investment during 1960-1990. Secondly, the legislation in Japan has introduced numerous documents and legislation over the last decade to ensure the safety of the bicycle riding environment [48]. Moreover, the Japanese have a low-risk tolerance and a strong emphasis on protecting others and are less optimistic about crashes than Americans [23], which results in a strong emphasis on driver control. In comparison, although the US has a long history of motorization, micromobility has impacted road injuries, which may be for the following reasons: Firstly, varied state restrictions have resulted in a patchwork of safety records. Secondly, Americans prioritize personal freedom, which discourages restrictions that limit driver conduct, making US lawmakers more aggressive in demanding safety equipment in cars to safeguard drivers rather than restrict drivers’ behaviors [41].

China and India are seeing rapid urbanization and a growth in car traffic, despite relatively poor road infrastructure and roadways. China has more tightly controlled route planning and has invested enormous sums compared to India, resulting in a much better roadway. However, China also saw a much more rapid growth speed for markets in cars and two-wheelers from 1990 to 2005 [21], which explains the continuously escalating incidence risks for age, period, and cohort effects. Moreover, the tendency to “scramble” also leads to collisions, especially around intersections. In recent years, India has shown tamed risks. However, the incidence rate in India is already high. Poor road infrastructure contributes to the difficulty of reducing collisions, as does the tendency for vehicles to be over-occupied. Furthermore, because both countries have a young motorization history, inexperienced drivers and road users may commit violations and engage in risky behavior.

This research has limitations in three aspects. In terms of micromobility definition, the boundaries between whether a micromobility device is categorized as “bicycle” or “motorcycle” are blurred within and between countries, thus making it difficult to conclude which type of micromobility device has a more significant impact on road collisions. On the data aspect, the GBD 2019 data only covers the years 1990 to 2019, while the new implementation of the ICD code was launched in October 2020 [15]. We cannot guarantee that the motorcyclist and cyclist injury data provided by GBD contains all fatalities and events involving micromobility due to the ambiguous definition of a micromobility device and the matching ICD number for micromobility-related injuries. For the method aspect, though we analyzed the age, period, and cohort effects separately, the influencing factors such as environmental development, road infrastructure, and vehicle legislation may coincide, making it unclear which has the most impact.

## Conclusions

This study evaluated the general mortality and incidence trends among road injuries, motorcyclist injuries, and cyclist injuries in China, India, Japan, and the US from 1990 to 2019 and estimated the contributions of age, period, and cohort effects to the trends. The overall ASMR, period, and cohort impacts improved overtime for road injury mortality. However, motorcyclist and cyclist injury mortality is inclined in China, India, and the US, consistent with the widespread trend in shared micromobility. Additionally, in recent years, ASIRs, period, and cohort effects have revealed higher hazards in China, India, and the US, particularly for micromobility-related injury occurrences. In Japan and the US, age groups before 30 and after 60 had a higher risk of all three categories of injuries, but China and India had a higher risk for populations over 60. All countries reported an increasing number of older people involved in micromobility collisions, prompting action on micromobility standards, legislation, and regulation. For future research, it is essential to identify the consequences of specific road injury affecting factors and micromobility injuries.

## Supplementary Information

**Additional file 1.**

## Data Availability

The datasets generated and analyzed during the current study are available in the GBD Results Tool, http://ghdx.healthdata.org/gbd-results-tool [49].
