# Correction to: Demographics of road injuries and micromobility injuries among China, India, Japan, and the United States population: evidence from an age-period-cohort analysis

**DOI:** 10.1186/s12889-022-13255-0

**Published:** 2022-04-28

**Authors:** Yudi Zhao, Jinhong Cao, Yudiyang Ma, Sumaira Mubarik, Jianjun Bai, Donghui Yang, Kai Wang, Chuanhua Yu

**Affiliations:** Department of Epidemiology and Biostatistics, School of Public Health, Wuhan University, Wuhan, China

**Correction to: BMC Public Health 22, 760 (2022)**

**https://doi.org/10.1186/s12889-022-13152-6**

In the original publication [1] there was an incorrect funding acknowledgement. In this correction article the correct and incorrect funding acknowledgement are published. The original article has been updated.

**Incorrect funding**

- This research does not receive any funding.

**Correct funding**

- This work was funded by the National Natural Science Foundation of China (Grant No. 82173626, 81773552) and the National Key Research and Development Program of China (Grant No. 2018YFC1315302).

## References

[1] Zhao Y (2022). Demographics of road injuries and micromobility injuries among China, India, Japan, and the United States population: evidence from an age-period-cohort analysis. BMC Public Health..

