# Supplementary material for: Demographics of road injuries and micromobility injuries among China, India, Japan, and the United States population: evidence from an age-period-cohort analysis
Source: BMC Public Health. 2022 Apr 14;22:760. doi: 10.1186/s12889-022-13152-6 (PMC9011927; doi:10.1186/s12889-022-13152-6)
Supplement: Supplementary file 1 — Additional file 1. [file 12889_2022_13152_MOESM1_ESM.pdf]

Table 1. GBD ICD-10 code definition of road injury, motorcyclist injury and cyclist injury

Cause	ICD10
Road injuries	V01-V04.99, V06-V80.929, V82-V82.9, V87.2-V87.3
Cyclist road injuries	V10-V19.9
Motorcyclist road injuries	V20-V29.9

Table 2. The Wald Chi-Square tests for estimable functions in the APC model

Null Hypothesis	Road Injury Deaths Chi-Square	Road Injury Deaths *P*-Value	Road Injury Incidence Chi-Square	Road Injury Incidence *P*-Value	Motorcyclist Injury Deaths Chi-Square	Motorcyclist Injury Deaths *P*-Value	Motorcyclist Injury Incidence Chi-Square	Motorcyclist Injury Incidence *P*-Value	Cyclist Injury Deaths Chi-Square	Cyclist Injury Deaths *P*-Value	Cyclist Injury Incidence Chi-Square	Cyclist Injury Incidence *P*-Value
**China**												
NetDrift = 0	416.84	<0.001	2237.11	<0.001	719.42	<0.001	742.07	<0.001	64.75	<0.001	1471.68	<0.001
All Period RR = 1	266.62	<0.001	2645.89	<0.001	282.96	<0.001	1172.5	<0.001	286.93	<0.001	1856.02	<0.001
All Cohort RR = 1	123.35	<0.001	2656.03	<0.001	95.98	<0.001	1194.68	<0.001	103.48	<0.001	2102.88	<0.001
All Local Drifts = Net Drift	88.64	<0.001	74.78	<0.001	87.6	<0.001	91.91	<0.001	93.06	<0.001	75.33	<0.001
**India**												
NetDrift = 0	1377.83	<0.001	614.28	<0.001	2785.85	<0.001	777.01	<0.001	537.63	<0.001	793.74	<0.001
All Period RR = 1	93.78	<0.001	123.73	<0.001	159.22	<0.001	258.85	<0.001	109.3	<0.001	127.11	<0.001
All Cohort RR = 1	269.61	<0.001	124.47	<0.001	198.8	<0.001	245.81	<0.001	184.41	<0.001	107.99	<0.001
All Local Drifts = Net Drift	219.16	<0.001	57.03	<0.001	102.88	<0.001	47.15	<0.001	131.92	<0.001	65.58	<0.001
**Japan**												
NetDrift = 0	4938.72	<0.001	7731.03	<0.001	1795.51	<0.001	6867.24	<0.001	432.37	<0.001	4526.01	<0.001
All Period RR = 1	5033.86	<0.001	6193.34	<0.001	415.37	<0.001	2459.65	<0.001	477.83	<0.001	4121.57	<0.001
All Cohort RR = 1	6729.51	<0.001	6549.78	<0.001	1804.93	<0.001	3844.71	<0.001	1283.5	<0.001	4670.65	<0.001
All Local Drifts = Net Drift	90.6	<0.001	98.87	<0.001	260.96	<0.001	377.21	<0.001	14.61	0.48	292.92	<0.001
**The US**												
NetDrift = 0	3835	<0.001	1083.11	<0.001	681.55	<0.001	453.45	<0.001	384.63	<0.001	209.88	<0.001
All Period RR = 1	701.68	<0.001	52.54	<0.001	153.06	<0.001	73.87	<0.001	46.71	<0.001	64.21	<0.001
All Cohort RR = 1	1117.27	<0.001	266.59	<0.001	303.45	<0.001	247.34	<0.001	355.33	<0.001	297.82	<0.001
All Local Drifts = Net Drift	266.74	<0.001	172.4	<0.001	236.74	<0.001	231.41	<0.001	307.86	<0.001	237.18	<0.001

Table 3. Overall annual percent change values for road injury, motorcyclist injury, and cyclist injury mortality and incidence in China, India, Japan, and the US

			Net Drift (%/year)	CI Lo	CI Hi
**Road injury**	**Mortality**	**China**	-0.443	-0.611	-0.275
		**India**	-0.433	-0.563	-0.304
		**Japan**	-4.692	-4.822	-4.563
		**USA**	-1.577	-1.696	-1.457
	**Incidence**	**China**	2.480	2.380	2.581
		**India**	1.018	0.726	1.312
		**Japan**	-2.485	-2.547	-2.422
		**USA**	-0.498	-0.692	-0.304
**Motorcyclist injury**	**Mortality**	**China**	0.144	-0.150	0.438
		**India**	0.653	0.466	0.840
		**Japan**	-4.285	-4.700	-3.868
		**USA**	2.027	1.629	2.426
	**Incidence**	**China**	3.025	2.847	3.204
		**India**	1.936	1.633	2.240
		**Japan**	-2.434	-2.532	-2.337
		**USA**	0.956	0.708	1.204
**Cyclist injury**	**Mortality**	**China**	0.018	-0.191	0.227
		**India**	-0.583	-0.758	-0.408
		**Japan**	-4.715	-5.133	-4.295
		**USA**	-0.589	-0.903	-0.274
	**Incidence**	**China**	2.550	2.429	2.672
		**India**	0.736	0.399	1.074
		**Japan**	-2.257	-2.327	-2.187
		**USA**	0.236	-0.086	0.558
